# Supplementary material for: Endogenous modulation of human visual cortex activity improves perception at twilight
Source: Nat Commun. 2018 Apr 10;9:1274. doi: 10.1038/s41467-018-03660-8 (PMC5893589; doi:10.1038/s41467-018-03660-8)
Supplement: Supplementary file 1 — Supplementary Information(PDF 2205 kb) [file 41467_2018_3660_MOESM1_ESM.pdf]

Supplementary Information

for

Endogenous Modulation of Human Visual Cortex Activity Improves Perception at Twilight

Lorenzo Cordani, Enzo Tagliazucchi, Céline Vetter, Christian Hassemer, Till Roenneberg, Jörg H. Stehle, Christian A. Kell

This PDF file includes:

Supplementary Figure 1

Supplementary Figure 2

Supplementary Table 1

Supplementary Table 2

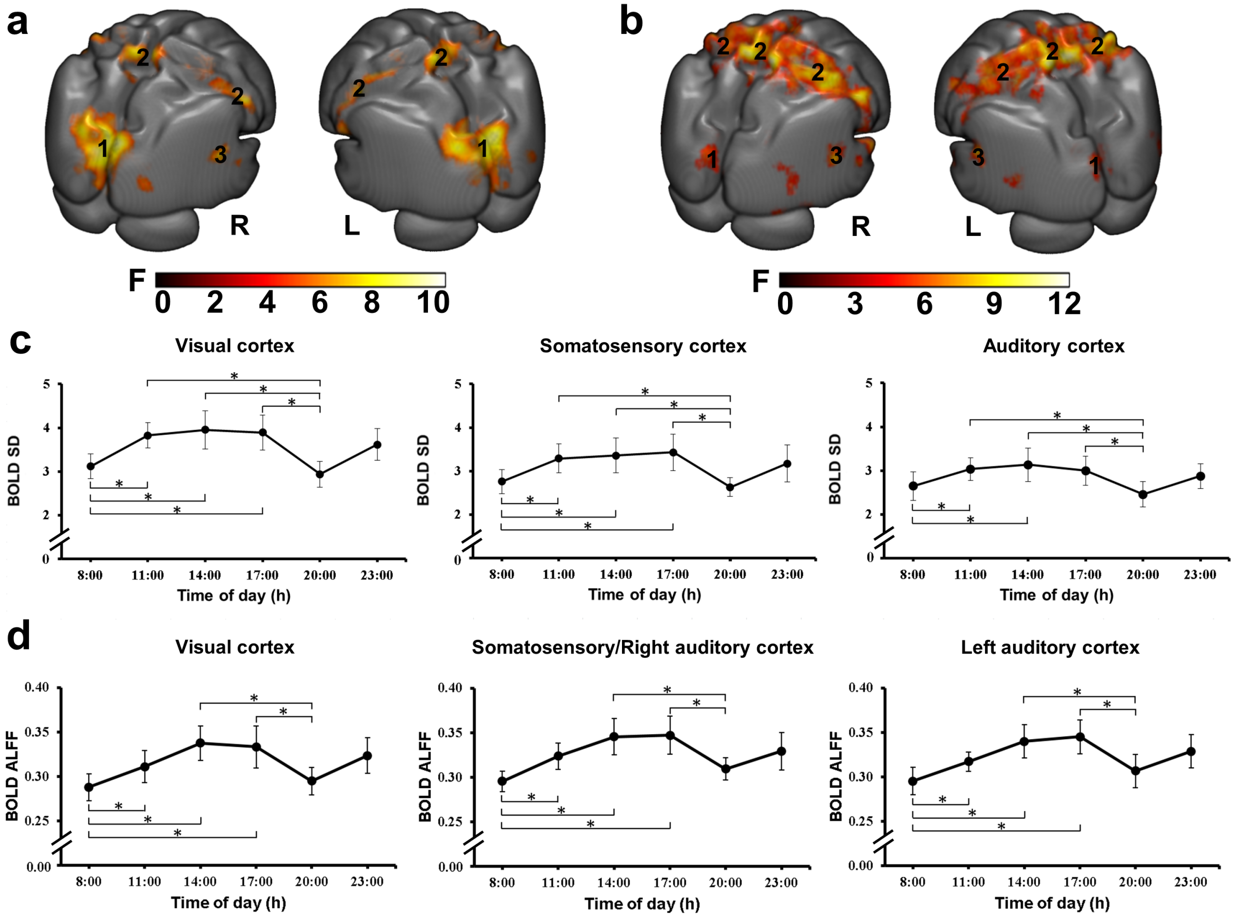

Supplementary Fig. 1 The ToD patterns of resting-state BOLD ALFF and BOLD SD are analogous, indicating that BOLD SD is unbiased by non-neural artifacts.

For visualization purposes, main effects of resting-state BOLD SD (**a**) and resting-state BOLD ALFF (**b**) were rendered on a group-averaged high-resolution T1 image. Color indicates F values. Like its BOLD SD counterpart, BOLD ALFF showed a significant main effect of ToD in the repeated measures ANOVA in bilateral visual (marked 1 in a and b), bilateral somatosensory (marked 2 in a and b), and right auditory and left auditory cortices (marked 3 in a and b) (cluster-extent based threshold: k = 269, $P < 0.05$, FWE; cluster-defining primary threshold of $F(5, 65) > 4.70$, $P < 0.001$; for details on local maxima see Supplementary Table 1). Significant clusters around the central sulcus were more extensive for BOLD ALFF and extended over primary somatosensory and motor cortices, confirming tight sensorimotor interactions in the Rolandic region[1, 2, 3]. In contrast, significant ToD-effects in visual cortex were more widespread for BOLD SD.

c The left panel recapitulates resting-state BOLD SD group analysis results in the visual cortex (Fig. 1b in the main paper). Somatosensory (middle panel) and auditory (right panel) cortices show analogous decreases in resting-state BOLD SD at 08:00 h and 20:00 h compared to midday measurements (graphs show the group-mean BOLD SD values and error bars represent the 95% confidence interval. Asterisks mark significant pairwise differences at $P < 0.05$, Bonferroni corrected). For further statistical details see Table 2 in the main paper.

d Likewise to results obtained for BOLD SD (**c**), group analysis of resting-state BOLD ALFF show significant decreases in bilateral visual (left panel), bilateral somatosensory and right auditory (middle panel), and left auditory cortices (right panel) at 08:00 h and 20:00 h compared to midday measurements (graphs show the group-mean BOLD ALFF values and error bars represent the 95% confidence interval. Asterisks mark significant pairwise differences at $P < 0.05$). For statistics see Supplementary Table 2.

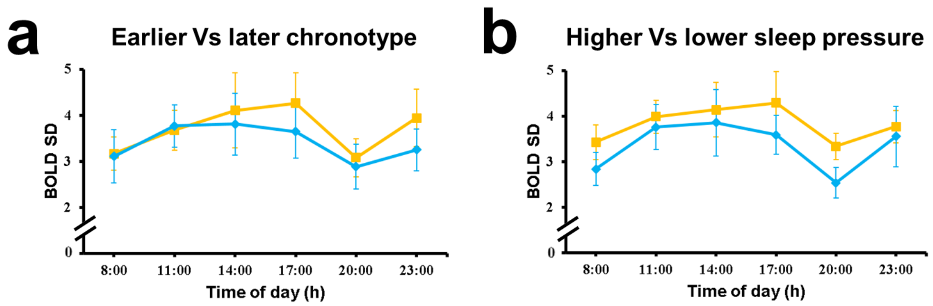

Supplementary Fig. 2 Illustrations of interactions between ToD and chronotype as well as between ToD and sleep pressure.

We tested resting-state BOLD SD for interactions between ToD and chronotype, sleep pressure, and sleep debt using the linear mixed model that also accounted for scanning day, heart and respiratory rate, body temperature, subjective sleepiness, movement during scanning (see main paper). While the main effect of ToD remained significant, there was a significant interaction between ToD and chronotype ($F(5, 34.603) = 2.738$, $P = 0.035$, type III F-test, $n = 14$) and a significant interaction between ToD and sleep pressure ($F(5, 32.21) = 5.128$, $P = 0.001$, type III F-test, $n = 14$). For visualization purposes, the graph plots group-mean resting-state BOLD SD values for participants with earlier (yellow) and later (blue) chronotypes (**a**) and for participants with higher (yellow) and lower (blue) sleep pressure (**b**). Groups were defined by a median split. Earlier chronotypes and participants with higher sleep pressure had higher BOLD SD values than later chronotypes and participants with lower sleep pressure in the second half of the day. There was no significant interaction between ToD and sleep debt ($F(5, 38.554) = 1.922$, $P = 0.113$, type III F-test, $n = 14$).

Anatomical region	Cluster p-value (FWE)	Cluster size (in voxels)	Local maxima	MNI coordinates (x,	y,	z)
Right and left rolandic cortex (Sensorimotor cortex)	0.001	13726	R Paracentral Lobule	6	-36	66
			R Paracentral Lobule	4	-32	62
			R Postcentral Gyrus	58	-8	40
			R Paracentral Lobule	10	-40	68
			L Precuneus	-12	-40	68
			R Precentral Gyrus	34	-22	50
			L Postcentral Gyrus	-44	-18	56
			R Postcentral Gyrus	54	-8	32
			L Paracentral Lobule	-8	-34	62
			L Precentral Gyrus	-18	-18	70
			L Postcentral Gyrus	-44	-20	52
			R Precentral Gyrus	40	-24	54
			R Postcentral Gyrus	46	-26	52
Right lateral temporal cortex (Auditory cortex)			R Superior Temporal Gyrus	66	-6	2
			R Superior Temporal Gyrus	56	-46	22
			R Middle Temporal Gyrus	64	-50	14
Right occipital cortex (Visual cortex)	0.001	2301	R Cuneus	22	-70	22
			R Cuneus	22	-72	26
			R Cerebellum	8	-52	-10
			R Posterior Cingulate Cortex	6	-40	10
			R Calcarine Gyrus	6	-74	10
			R Posterior Cingulate Cortex	10	-40	12
			R Precuneus	10	-46	8
			R Lingual Gyrus	22	-72	-4
			R Calcarine Gyrus	20	-68	-18
			R Lingual Gyrus	22	-54	-4
			R Precuneus	12	-46	12
			R Lingual Gyrus	22	-74	0
			R Lingual Gyrus	16	-50	2
			R Lingual Gyrus	24	-64	-2
Left occipital cortex (Visual cortex)	0.001	1912	L Calcarine Gyrus	-24	-64	6
			L Lingual Gyrus	-18	-70	-4
			L Lingual Gyrus	-12	-70	4
			L Calcarine Gyrus	-18	-70	8
			L Calcarine Gyrus	-16	-76	14
			L Posterior Cingulate Cortex	-14	-42	8
			L Precuneus	-22	-52	4
			L Lingual Gyrus	-26	-60	-2
			L Superior Occipital Gyrus	-10	-94	20
			L Cuneus	-14	-84	16
			L Precuneus	-18	-46	6
			L Lingual Gyrus	-16	-78	-10
			L Lingual Gyrus	-14	-60	-10
			L Superior Occipital Gyrus	-14	-80	26
Left lateral temporal cortex (Auditory cortex)	0.001	475	L Superior Temporal Gyrus	-64	-40	18
			L Superior Temporal Gyrus	-52	-36	12
			L Superior Temporal Gyrus	-48	-40	20
			L Superior Temporal Gyrus	-66	-46	12
			L Superior Temporal Gyrus	-44	-36	22
			L Middle Temporal Gyrus	-50	-48	22
			L Superior Temporal Gyrus	-44	-38	10
Left lateral temporal cortex (Auditory cortex)	0.001	355	L Middle Temporal Gyrus	-46	-14	-22
			L Middle Temporal Gyrus	-50	-18	-14
			L Middle Temporal Gyrus	-48	-10	-20
			L Middle Temporal Gyrus	-64	-4	-12
			L Middle Temporal Gyrus	-58	-12	-20
			L Middle Temporal Gyrus	-56	-10	-8
			L Middle Temporal Gyrus	-54	-18	-22
			L Superior Temporal Gyrus	-46	-20	-6
Left lateral temporal cortex (Auditory cortex)	0.001	269	L Middle Temporal Gyrus	-48	-56	8
			L Middle Temporal Gyrus	-50	-68	8
			L Middle Temporal Gyrus	-44	-58	10
			L Middle Temporal Gyrus	-50	-56	4

Supplementary Table 1 Brain regions showing ToD-effects of BOLD ALFF.

Denomination of local maxima was adopted from the SPM Anatomy toolbox[4]. MNI-coordinates represent peak activations inside clusters.

Anatomical region	t-test	t(13)	*P*-value	Cohen's *d*
**Visual cortex**	**11:00h–08:00h**	2.89	0.013	0.72
	**14:00h–08:00h**	6.17	<0.001	1.47
	**17:00h–08:00h**	5.13	<0.001	1.11
	**11:00h–20:00h**[a]	1.44	0.173	0.50
	**14:00h–20:00h**	4.00	0.002	1.27
	**17:00h–20:00h**	3.47	0.004	0.98
**Somatosensory cortex, right auditory cortex**	**11:00h–08:00h**	4.28	0.001	1.10
	**14:00h–08:00h**	7.23	<0.001	1.35
	**17:00h–08:00h**	6.19	<0.001	1.42
	**11:00h–20:00h**[a]	1.49	0.160	0.55
	**14:00h–20:00h**	3.86	0.002	1.09
	**17:00h–20:00h**	3.66	0.003	1.11
**Left auditory cortex**	**11:00h–08:00h**	3.15	0.008	0.83
	**14:00h–08:00h**	5.15	<0.001	1.36
	**17:00h–08:00h**	6.41	<0.001	1.47
	**11:00h–20:00h**[a]	0.98	0.345	0.36
	**14:00h–20:00h**	2.96	0.011	0.94
	**17:00h–20:00h**	3.36	0.005	1.07

Supplementary Table 2 Repeated measures ANOVA post-hoc t-tests on resting-state BOLD ALFF.

Planned pairwise comparisons for BOLD ALFF (planned based on the results of the analysis of BOLD SD) for each cluster (significance threshold at $P < 0.05$).

[a] Marked t-tests were subthreshold, but listed in the table for completeness. All other comparisons were $P < 0.05$.

Supplementary References

1. Uematsu S., Lesser R. P., Gordon B. Localization of sensorimotor cortex: the influence of Sherrington and Cushing on the modern concept. *Neurosurgery* **30,** 904-912 (1992).

2. Nii Y., Uematsu S., Lesser R. P., Gordon B. Does the central sulcus divide motor and sensory functions? Cortical mapping of human hand areas as revealed by electrical stimulation through subdural grid electrodes. *Neurology* **46,** 360-367 (1996).

3. Moore C. I., et al. Segregation of somatosensory activation in the human rolandic cortex using fMRI. *J. Neurophysiol.* **84,** 558-569 (2000).

4. Eickhoff S. B., et al. A new SPM toolbox for combining probabilistic cytoarchitectonic maps and functional imaging data. *Neuroimage* **25**, 1325-1335 (2005).
